# The prognostic value of the GPAT/AGPAT gene family in hepatocellular carcinoma and its role in the tumor immune microenvironment

**DOI:** 10.3389/fimmu.2023.1026669

**Published:** 2023-02-10

**Authors:** Peizhen Wen, Rui Wang, Yiqun Xing, Wanxin Ouyang, Yixin Yuan, Shuaishuai Zhang, Yuan Liu, Zhihai Peng

**Affiliations:** ^1^ Organ Transplantation Clinical Medical Center of Xiamen University, Department of Organ Transplantation, Xiang’an Hospital of Xiamen University, School of Medicine, Xiamen University, Xiamen, Fujian, China; ^2^ Organ Transplantation Institute of Xiamen University, Fujian Provincial Key Laboratory of Organ and Tissue Regeneration, School of Medicine, Xiamen University, Xiamen, Fujian, China; ^3^ Department of General Surgery, Shanghai General Hospital, Shanghai Jiao Tong University, Shanghai, China

**Keywords:** HCC, GPAT/AGPAT, LASSO-Cox analysis, nomogram, immune infiltration

## Abstract

**Background:**

Liver cancer is the sixth most commonly diagnosed cancer and the third leading cause of cancer-related death worldwide. Hepatocellular carcinoma accounts for an estimated 90% of all liver cancers. Many enzymes of the GPAT/AGPAT family are required for the synthesis of triacylglycerol. Expression of AGPAT isoenzymes has been reported to be associated with an increased risk of tumorigenesis or development of aggressive phenotypes in a variety of cancers. However, whether members of the GPAT/AGPAT gene family also influence the pathophysiology of HCC is unknown.

**Methods:**

Hepatocellular carcinoma datasets were obtained from the TCGA and ICGC databases. Predictive models related to the GPAT/AGPAT gene family were constructed based on LASSO-Cox regression using the ICGC-LIRI dataset as an external validation cohort. Seven immune cell infiltration algorithms were used to analyze immune cell infiltration patterns in different risk groups. IHC, CCK-8, Transwell assay, and Western blotting were used for in vitro validation.

**Results:**

Compared with low-risk patients, high-risk patients had shorter survival and higher risk scores. Multivariate Cox regression analysis showed that risk score was a significant independent predictor of overall survival (OS) after adjustment for confounding clinical factors (p < 0.001). The established nomogram combined risk score and TNM staging to accurately predict survival at 1, 3, and 5 years in patients with HCC with AUC values of 0.807, 0.806, and 0.795, respectively. This risk score improved the reliability of the nomogram and guided clinical decision-making. In addition, we comprehensively analyzed immune cell infiltration (using seven algorithms), response to immune checkpoint blockade, clinical relevance, survival, mutations, mRNA expression-based stemness index, signaling pathways, and interacting proteins related to the three core genes of the prognostic model (AGPAT5, LCLAT1, and LPCAT1). We also performed preliminary validation of the differential expression, oncological phenotype, and potential downstream pathways of the three core genes by IHC, CCK-8, Transwell assay, and Western blotting.

**Conclusion:**

These results improve our understanding of the function of GPAT/AGPAT gene family members and provide a reference for prognostic biomarker research and individualized treatment of HCC.

## 1 Introduction

In 2020, liver cancer was the sixth most prevalent cancer and the third leading cause (8.3%) of cancer-related death worldwide, after lung cancer (18%) and colorectal cancer (9.4%) (1–3). Hepatocellular carcinoma (HCC) is the most common type of liver cancer, accounting for approximately 90% of all liver cancer cases (2). Despite the progress in the diagnosis and treatment of HCC over recent years, the treatment of which still faces considerable challenges. The identification of more effective therapeutic targets and more promising prognostic biomarkers is essential for the control of liver cancer globally. There is also an urgent need for optimal stratification of patients with HCC to allow the adequate monitoring of patients with different degrees of malignancy and, consequently, the implementation of more precise diagnostic and therapeutic measures.

Physiological lipid metabolism represents an alternative source of energy and has been widely demonstrated to play an important role in microenvironmental adaptation and cellular signaling. However, dysregulated lipid metabolism has also been implicated in the development and progression of HCC (4–6). Recently, many enzymes of the GPAT/AGPAT family have been identified (7, 8). 1-Acylglycerol-3-phosphate *O*-acyltransferases (AGPATs) is essential for the production of triacylglycerol (TAG), and these enzymes are also involved in the synthesis of most fatty acids (7). AGPAT isozymes reportedly promote proliferation and drug resistance in cancer cells and are associated with a high risk of tumor formation or aggressive tumor profiles (9–12).

The tumor microenvironment (TME) contains a wide variety of immune cells that have complex interactions and regulation with tumor cells. Understanding the abundance of immune cells in tumor samples is valuable for the discovery of tumor immunotherapeutic agents and clinical decision-making in therapeutic regimens (13). The correlation between the level of tumor immune cell infiltration and clinical prognosis has been studied in many cancers (14–19).

In this study, we integrated clinical information and gene expression profile data for HCC patients from The Cancer Genome Atlas (TCGA) database. A risk prediction model based on the *GPAT*/*AGPAT* gene family was constructed based on a least absolute shrinkage and selection operator (LASSO)-Cox regression, with the ICGC-LIRI dataset serving as the external validation cohort. Subsequently, a nomogram was plotted for predicting 1-, 3-, and 5-year overall survival (OS). We also compared immune cell infiltration in different risk populations using multiple cutting-edge algorithms and performed a comprehensive bioinformatic analysis of the three core genes of the predictive model, as well as a preliminary validation of the oncological phenotype. In conclusion, we established a novel prognostic signature for HCC based on the *GPAT*/*AGPAT* gene family and performed an in-depth assessment of the potential biological functions of this gene family in HCC. The results enhance our understanding of the function of the *GPAT*/*AGPAT* gene family and provide prognostic biomarkers that may aid in the development of individualized treatments for HCC.

## 2 Materials and methods

### 2.1 Data source

RNA sequencing expression profiles (level 3), gene mutations, and associated clinical data were collected from TCGA (https://portal.gdc.cancer.gov/), GTEx (https://gtexportal.org/home/datasets), and ICGC (https://dcc.ICGC.org/releases) databases. Pan-cancer data were retrieved from the UCSC Xena Browser (http://xena.ucsc.edu/). All data were filtered to remove duplicate records before being normalized using Log2(TPM+1) transformation. As the data used in this study were gathered from public databases, approval from an ethics committee was not required.

### 2.2 Gene expression analysis

The expression profiles of genes belonging to the *GPAT*/*AGPAT* gene family were extracted from 531 cases of RNA sequencing data from TCGA-GTEx-LIHC after normalization by Log2(TPM+1) transformation. The Mann-Whitney U test was employed to assess *GPAT*/*AGPAT* gene expression in unpaired tumor and normal tissue samples. The Wilcoxon signed-rank test was utilized to assess the expression of the genes in paired tumor and normal tissue samples. The Kruskal-Wallis test was used to compare the expression of the genes in tumor and normal tissue samples grouped by different clinical variables. All statistical analysis was performed using R (version 3.6.3). Plots were generated using the “ggplot2” package. *P*-values<0.05 were considered significant.

### 2.3 The human protein atlas

The HPA is a proteomics-based database that contains the antibody-based expression profiles of proteins, allowing the assessment of protein expression (immunohistochemical results) in different tumors as well as in the corresponding normal tissues.

### 2.4 Construction of the GPAT/AGPAT family-related gene prognostic signature

Using the TCGA-LIHC cohort (*n* = 371), the “survival” R package was used to run a univariate Cox analysis on *GPAT*/*AGPAT* family-related genes to uncover OS-associated mRNAs. Subsequently, patients were randomly divided into training and validation sets in a 1:1 ratio, and the R package “glmnet” was used to conduct a LASSO Cox regression analysis in the training set to obtain a coefficient for each OS-related mRNA. To prevent overfitting, a 10-fold cross-validation was performed. the penalized regularization parameter λ was selected by the cross-validation program cv.glmnet, “nfolds = 10”, and lambda.min was used to determine the λ value. The risk score of each sample was then calculated according to the following formula: $\sum_{i=1}^{n}\left(Expi*Coefi\right)$ , where Coef refers to the coefficient of survival-related mRNAs and Exp denotes mRNA expression. Samples from the TCGA-LIHC training set were separated into high-risk and low-risk groups according to the median risk score of each sample.

### 2.5 Validation of the model

Kaplan-Meier survival analysis was used to analyze the differences in OS between the high-risk and low-risk groups in the training, validation, and total sets of TCGA-LIHC using the R packages “survminer” and “survivor”. The sensitivity and specificity of this prognostic model were assessed by plotting the receiver operating characteristic (ROC) curve and area under the curve (AUC) using the R packages “timeROC” and “ggplot2”. Further validation was performed in the ICGC-LIRI external validation set. To investigate the effect of the specified variables on OS, univariate and multivariate Cox regression analysis was undertaken in the R package “survival”. Finally, the R packages “rms” and “survival” were used to build the nomogram of the multivariate model, and a calibration plot was used to evaluate the predictive ability of the nomogram.

### 2.6 Analysis of immune cell infiltration

Immuno-infiltration algorithms were derived from the R packages “GSVA” (ssGSEA) (20) and “immunedeconv” (21) [TIMER (22), xCell (23), MCP-counter (24, 25), CIBERSORT (26), EPIC (27), and quanTIseq (28)]. Enrichment scores were calculated for each sample using the ssGSEA algorithm based on the 24 immune cell markers provided by Bindea et al (29). Finally, the Mann-Whitney U test (Wilcoxon rank sum test) was used to determine the difference in immune cell infiltration between the high-risk and low-risk groups. Significance signs: ns, p ≥ 0.05; *, p< 0.05; **, p< 0.01; ***, p< 0.001.

### 2.7 Mutation analysis

Based on the expression of the *AGPAT5*, *LCLAT1*, and *LPCAT1* genes, the 369 samples from TCGA-LIHC with detected mutations were separated into high- and low-expression groups, and the differences in mutation frequencies in each group were examined using chi-square tests. The mutation landscape of the 20 genes with the highest mutation frequencies was presented as waterfall plots using the R package “maftools”. The *AGPAT5*, *LCLAT1*, and *LPCAT1* mutation types in HCC were further evaluated using the Catalogue of Somatic Mutations in Cancer (COSMIC) database (http://cancer.sanger.ac.uk). We integrated sample gene expression data and TMB, MSI, purity, and HRD data to calculate the Pearson correlation between genomic heterogeneity indicators and gene expression. The Simple Nucleotide Variation dataset of the level4 of all TCGA samples processed by MuTect2 software (30) was downloaded from GDC (https://portal.gdc.cancer.gov/), and the tmb function of the r package “maftools” was used to calculate the TMB (Tumor mutation burden) of each tumor. MSI (microsatellite instability) score of each tumor obtained from the previous study (31). Tumor purity data and tumor HRD (homologous recombination deficiency) data were obtained from a previous study (32).

### 2.8 Correlation analysis

We obtained common tumor-associated pathway gene sets from published studies and MsigDB database (http://www.broadinstitute.org/gsea/msigdb/index.jsp), such as Cellular_response_to_hypoxia, Tumor_proliferation_signature, EMT_markers, ECM-relatted_genes, Angiogenesis, Apoptosis, DNA_repair, G2M_checkpoint, Inflammatory_response, PI3K_AKT_mTOR_pathway, P53_pathway, MYC_targets, TGFB, IL-10_Anti-inflammatory_Signaling_Pathway, Genes_up-regulated_by_reactive_oxigen_species_(ROS), DNA_replication, Collagen_formation, and Degradation_of_ECM. The absolute enrichment fraction of gene sets in individual samples was calculated using the R package “GSVA” with parameter method=“ssgsea”. The association between gene expression and pathway scores was assessed using Spearman’s rank-order correlation and visualized in R using the “ggplot2” package.

### 2.9 Protein-protein interaction network and drug sensitivity analysis

Data for constructing the PPI network were obtained from the STRING database (interaction score >0.7) and further visualized using the R packages “igraph” and “ggraph”. Gene Set Cancer Analysis (GSCA), a comprehensive cancer analysis database that can be utilized to study the link between mRNA expression and drug IC50 values, was used for drug sensitivity analysis. Raw data were primarily obtained from the Genomics of Drug Sensitivity in Cancer (GDSC) and Cancer Therapeutics Response Portal (CTRP) drug databases.

### 2.10 Immunohistochemistry

Immunohistochemistry was performed on paraffin-embedded sections of human hepatocellular carcinoma samples. Briefly, after deparaffinized and rehydrated, tissue sections were subjected to antigen repair in a microwave oven with EDTA antigen repair buffer (pH 9.0) for 8 min on medium heat until boiling, ceased for 8 min to hold and then turned to medium-low heat for 7 min. Endogenous peroxidase activity was blocked with 3% hydrogen peroxide solution for 25 min at room temperature. After washing, the non-specific binding sites were blocked by incubation with 3% BSA for 30 min at room temperature. Sections were then incubated with anti-AGPAT5 (Affinity Biosciences, DF3641, 1:100), anti-LCLAT1 (abcepta, AP5723b, 1:100) and anti-LPCAT1 (proteintech, 16112-1-AP, 1:300) overnight at 4°C. After incubation with the primary antibody, sections were washed and the tissue was covered with secondary antibody (HRP-labeled) of the species corresponding to the primary antibody and incubated at room temperature for 50 min. after which the sections were incubated with DAB staining reagent. After restaining with hematoxylin and dehydration, the sections were sealed and imaged using a Leica microscope. Tissue microarrays of human hepatocellular carcinoma and paired adjacent normal tissues (HLiv-HCC060PG-01) were purchased from Shanghai Outdo Biotech Company (Shanghai, China). The study was approved by the ethics committee of Shanghai Outdo Biotech Company.

### 2.11 Cell culture and siRNA transfection

The human liver cancer cell line HepG2 was obtained from Stem Cell Bank, Chinese Academy of Sciences (Shanghai, China). HepG2 cells were cultured in high-sugar DMEM supplemented with 10% fetal bovine serum (GeminiBio, 900-108-A46G00J, USA) and 1% penicillin and streptomycin, and all cells were incubated at 37°C in a humidified environment containing 5% CO2. siRNA-AGPAT5 (5′- GCCUGUGGGUUUACUAUUAATT-3′ forward, and 5′-UUAAUAGUAACCCACAGGCTT-3′ reverse), siRNA-LCLAT1 (5′-CAGCCACAUUUAAAUUCAATT-3′ forward, and 5′-UUGAAUUUAAAUGUGGCUGTT-3′ reverse), siRNA-LPCAT1 (5′-CCAGAAGGAACUUGUACAATT-3′ forward, and 5′-UUGUACAAGUUUCCUUCUGGTT-3′ reverse) and non-targeting control siRNA (NC-siRNA, 5′-UUCUCCGAACGUGUCACGUTT-3′ forward, and 5′-ACGUGACACGUUCGGAGAATT-3′ reverse) were obtained from GenePharma Ltd (Shanghai, China). Cells were transfected with Lipofectamine 3000 (Invitrogen, L3000015) according to the manufacturer’s instructions, and protein level changes were detected after 48 h.

### 2.12 Western blotting

After 48 h of transfection, cell lysates were obtained using RIPA lysis buffer (Solarbio Life Sciences, R0010, Beijing, China) and protease phosphatase inhibitor mixture (Beyotime Biotechnology, P1048, Shanghai, China), and total proteins were extracted. Total proteins were electrophoresed by SDS-PAGE gels (8%-10%) and then transferred to 0.45 µm PVDF membranes (Millipore, IPVH00010, Germany) and sealed with 5% skim milk for 120 min at room temperature. The PVDF membranes were then incubated overnight at 4°C with the primary antibody and the next day with the secondary antibody for 2 h at room temperature. Protein bands are detected with the ECL kit. The primary antibody against AGPAT5 was obtained from Affinity Biosciences (DF3641, USA). Primary antibody against LCLAT1 was obtained from abcepta (AP5723b, China). Primary antibodies against LPCAT1, Vimentin, E-Cadherin, GAPDH, Actin, and the corresponding species of secondary antibodies were obtained from proteintech (item numbers 16112-1-AP, 10366-1-AP, 20874-1-AP, 60004-1-lg, 66009-1-lg. SA00001-1 and SA00001-2, China). The primary antibody against VEGF was obtained from Santa Cruz Biotechnology (sc-7269, USA).

### 2.13 CCK-8

The transfected cells were inoculated in 96-well plates, and 5 replicate wells were set up in each group with 3000 cells/100 μL per well. the proliferation rate of HepG2 cells was detected by Cell Counting Kit-8 (MCE, HY-K0301-100T, USA). The absorbance of all wells at 450 nm was measured with an automated microplate reader at 24 h, 48 h, and 72 h, respectively.

### 2.14 Cell migration and invasion assay

*In vitro* migration and invasion assays were performed using PC polycarbonate membrane cell insert dishes (8.0-μm membrane, JET BIOFIL, TCS003024, China). Matrigel (Corning, 356230, USA) was wrapped around the membrane in the invasion assay. Post-transfected cells of logarithmic growth phase were taken and resuspended in serum-free medium, and the cell density was adjusted to 5*10^5/mL. 200 μL of cell suspension was inoculated in the upper chamber and 600 μL of medium supplemented with 10% FBS was added to the lower chamber. After 48-72 hours of incubation, the cells were fixed with 4% paraformaldehyde, and the cells in the upper chamber were gently wiped with cotton swabs and finally stained with 0.1% crystal violet for 15 minutes. Four to five × 200 magnification fields were randomly selected for cell counting. The experiment was repeated three times.

## 3 Results

### 3.1 Differential expression of *GPAT*/*AGPAT* gene family in HCC patients

**Figure 1:**
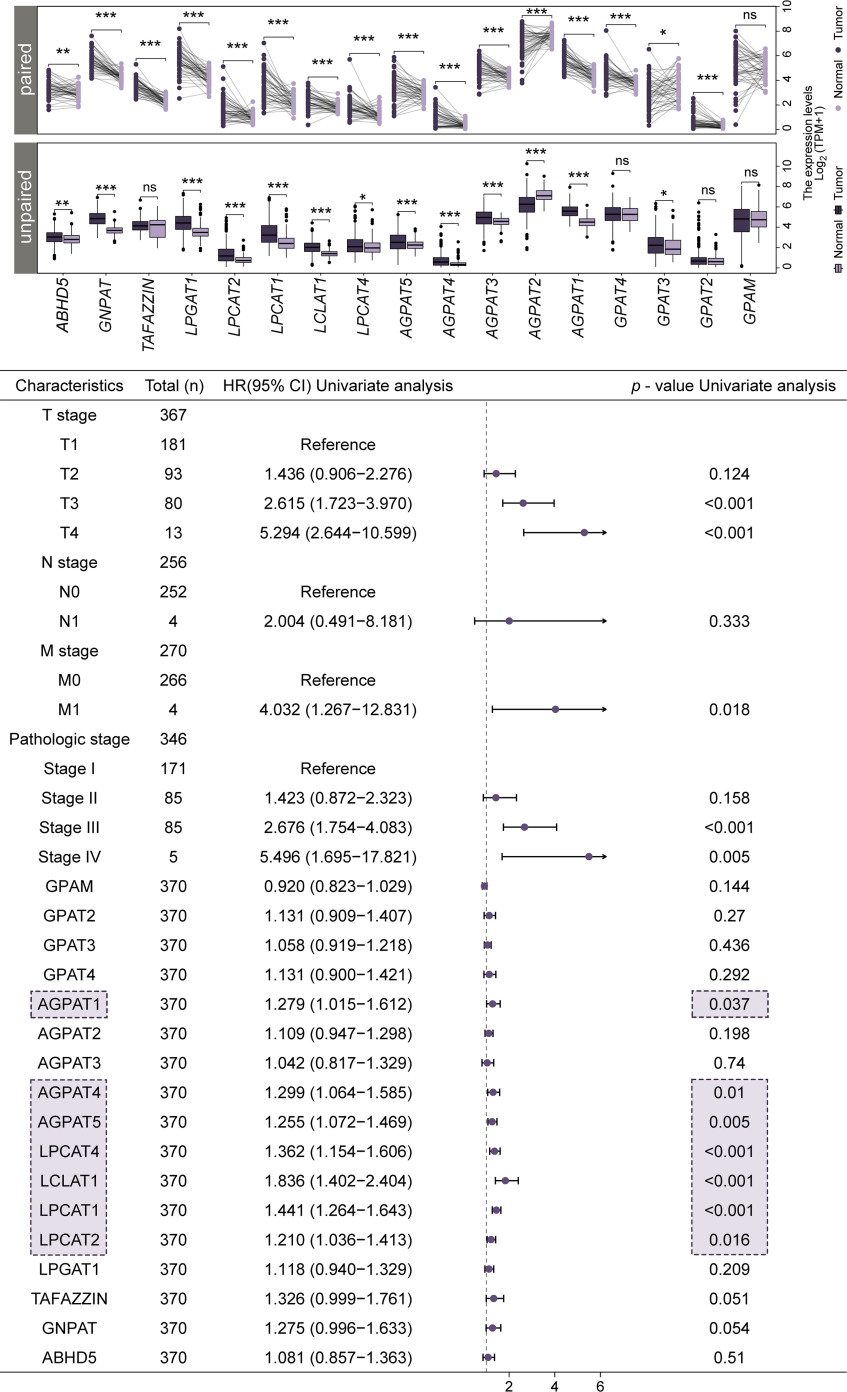
Differential expression of *GPAT*/*AGPAT* gene family members in HCC patients and forest plots from univariate Cox regression analysis. **(A)** Differential expression levels of 17 members of the *GPAT*/*AGPAT* gene family in paired and unpaired samples of TCGA-LIHC/GTEx. **(B)** Forest plot for univariate Cox regression analysis. Significance signs: ns, p ≥ 0.05; *p< 0.05; **p< 0.01; ***p< 0.001.

We analyzed the expression of 17 genes of the *GPAT*/*AGPAT* gene family in tissues of patients with LIHC and compared it with that in normal liver tissues. The samples consisted of 371 tumor tissues, 50 adjacent normal tissues, and 110 normal liver tissues. As shown in **Figure 1A**, the transcript levels of *GPAT3*, *AGPAT1*, *AGPAT3*, *AGPAT4*, *AGPAT5*, *LPCAT4*, *LCLAT1*, *LPCAT1*, *LPCAT2*, *LPGAT1*, *GNPAT*, and *ABHD5* were significantly upregulated in TCGA-LIHC unpaired samples compared with those in normal liver tissues, while the levels of *AGPAT2* were significantly decreased. These results are consistent with reports indicating that some members of the *GPAT*/*AGPAT* gene family are upregulated in multiple cancers and are associated with poor prognosis (33, 34). In TCGA-LIHC paired samples, the transcript levels of *GPAT2* and *GPAT4* were found to be significantly higher in tumor tissue samples than in the respective adjacent normal tissue samples (**Figure 1A**). We further retrieved the immunohistochemical staining data from the HPA database (**Supplementary Figure 1**) and found differential protein expression patterns similar to those obtained in the TCGA-GTEx-LIHC gene expression analysis.

### 3.2 The development of a prognostic model based on the *GPAT*/*AGPAT* gene family

**Figure 2:**
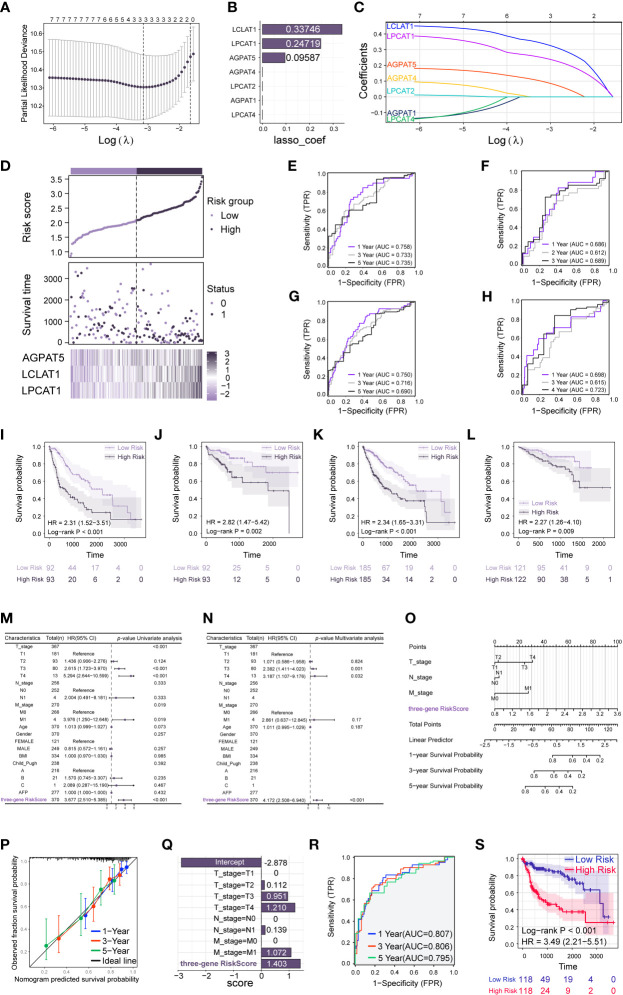
Construction and validation of prediction model based on the *GPAT*/*AGPAT* gene family. **(A, C)** LASSO penalized Cox regression was performed to screen out the three best prognosis-related genes. **(B)** Genes associated with overall survival (OS) and their coefficients. **(D)** Risk score distribution, risk grouping, survival outcome, and molecular expression for the prognostic model in the TCGA-LIHC training set. **(E–H)** Time-dependent receiver operating characteristic (ROC) analysis to assess the sensitivity and specificity of the prognostic model in TCGA training set **(E)**, validation set **(F)**, total set **(G)**, and ICGC-LIRI external validation set **(H)**. **(I–L)** Kaplan-Meier survival analysis comparing the differences in survival outcomes between the high-risk and low-risk groups in TCGA training set **(I)**, validation set **(J)**, total set **(K)**, and ICGC-LIRI external validation set **(L)**. **(M, N)** Univariate and multivariate Cox regression analysis identified the risk score as an independent prognostic factor when clinical variables were included. **(O)** A Cox model-based nomogram for predicting 1–5-year overall survival (OS). **(P)** Calibration curves of the nomogram in the overall set for predicting 1–5-year OS. **(Q)** Coefficients were obtained for each variable based on the results of the multivariable Cox model integrating the clinical variables and the three-gene risk score. **(R, S)** Time-dependent receiver operating characteristic (ROC) analysis **(R)** and Kaplan-Meier survival analysis **(S)** was performed based on the median grouping of the new risk scores for each sample.

To identify potential prognostic biomarkers for HCC among the genes in the GPAT/AGPAT family, we developed a prognostic risk model. First, we downloaded RNA sequencing data and clinical information for 371 tumor tissues and 50 adjacent normal tissues from the TCGA database and matched the sample IDs in the expression matrix with the clinical information. Then, we performed a univariate Cox regression analysis, incorporating TNM stage, pathologic stage, and expression values of *GPAT*/*AGPAT* gene family as variables. Seven genes were found to be significantly associated with OS (*p*< 0.05) (**Figure 1B**). The differential expression of these seven genes is shown in **Figure 1A**, and most of them were significantly associated with each other (**Supplementary Figure 2A**). Then, we randomly allocated 370 HCC patients (at a 1:1 ratio) into two groups, one for training and one for validation (*n* = 185 in each group). A LASSO Cox regression analysis (**Figure 2A**) was performed in the training set using the R package “glmnet” and a coefficient was generated for each OS-related gene (**Figure 2B**). The prognostic model consisted of 3 risk factors, namely, *AGPAT5*, *LCLAT1*, and *LPCAT1* (**Figure 2C**). For all samples, the risk score was calculated using the following formula: Risk score = (0.09587 × Exp of *AGPAT5*) + (0.33746 × Exp of *LCLAT1*) + (0.24719 × Exp of *LPCAT1*), where Exp denotes mRNA expression.

### 3.3 Validation of the prognostic model

All samples were grouped at the median according to the risk score of each sample. samples from the TCGA-LIHC training set, validation set, and total set were all categorized into high-risk and low-risk groups, respectively. Then, a Kaplan-Meier survival analysis was performed to examine whether there were differences in survival outcomes between the high-risk and low-risk groups. The results showed that patients in the high-risk group had a shorter median OS than those in the low-risk group (log-rank test, *p*< 0.001) (**Figure 2I**). Similar results were observed for the validation and total sets (*p* = 0.002 and *p*< 0.001, respectively) (**Figures 2J, K**). We also performed a time-dependent ROC analysis to determine the predictive accuracy of the model. For the training set, the AUC for 1-, 3-, and 5-year OS was 0.758, 0.733, and 0.735, respectively (**Figure 2E**); for the validation set, the AUC for 1-, 2-, and 3-year OS was 0.686, 0.612, and 0.689, respectively (**Figure 2F**); and for the total set, the AUC for 1-, 3-, and 5-year OS was 0.750, 0.716, and 0.690, respectively (**Figure 2G**). We further validated the ability of the model to predict OS using the ICGC-LIRI external validation set (*n* = 232). We calculated the risk score for each sample using the risk score formula mentioned above and divided the samples in the external validation set into high-risk and low-risk groups based on the median risk score. Then, we performed Kaplan-Meier survival analysis and time-dependent ROC analysis. The results showed that in the external validation set, the median OS was shorter in high-risk patients than in low-risk patients (log-rank test, *p* = 0.009) (**Figure 2L**). The AUC for OS at 1, 3, and 4 years was 0.698, 0.615, and 0.723, respectively (**Figure 2H**). The risk score distribution, risk grouping, survival outcome, and molecular expression of the prognostic model for the TCGA-LIHC training set are shown in **Figure 2D**; the same parameters for the TCGA-LIHC validation set, TCGA-LIHC total set, and the ICGC-LIRI external validation set are depicted in **Supplementary Figures 2B–D**, respectively. The data showed that the high-risk group had a lower survival rate and higher risk scores than the low-risk group. The heat maps illustrate the differential expression of risk factors between the two groups in each set. Finally, we performed univariate and multivariate Cox regression analyses using TCGA-LIHC data to investigate the relationship between clinical characteristics and the risk scores (**Figures 2M, N**). The variables included were TNM stage, age, gender, body mass index, Child_Pugh classification, alpha-fetoprotein level, and risk score. After adjusting for other confounding clinical characteristics, multivariate Cox regression analysis revealed that risk score was strongly linked with OS (*p*< 0.001), implying that risk score might serve as an independent prognostic factor for HCC (hazard ratio [HR] = 4.172, 95% CI = 2.508–6.940, *p*< 0.001). Similarly, in the ICGC-LIRI external validation dataset (**Supplementary Figures 2E, F**), the risk score remained an independent predictor of survival (HR = 2.314, 95% CI = 1.115–4.801, *p* = 0.024).

### 3.4 Nomogram construction

We constructed a nomogram for predicting 1–5-year OS (**Figure 2O**) based on the Cox model. After assigning scaled scores to the individual variables within the multivariate Cox regression model, total scores were calculated to predict the probability of event occurrence. Analysis of the calibration plot (**Figure 2P**) indicated that the nomogram was well-calibrated and that the average predicted probability for each subgroup was close to the observed probability. The concordance index for the model was 0.732 (0.698–0.766). Finally, we integrated the clinical variables and the three-gene risk score based on the results of the multivariate Cox model to obtain the coefficients for each variable (**Figure 2Q**). A new risk score was subsequently produced for each sample using the above-outlined procedure, followed by Kaplan-Meier survival analysis and time-dependent ROC analysis. The results showed that patients in the high-risk group had a shorter median OS than those in the low-risk group (log-rank test, *p*< 0.001) (**Figure 2S**). In TCGA total dataset, the AUC for 1-, 3-, and 5-year OS was 0.807, 0.806, and 0.795, respectively (**Figure 2R**). This implied that the multivariate Cox model, which integrated clinical variables and the three-gene risk score, exhibited higher predictive power, and further suggested that the GPAT/AGPAT gene family-related three-gene risk score prediction model is a stable and independent prognostic model for OS in HCC.

### 3.5 Immune cell infiltration patterns in different risk groups

**Figure 3:**
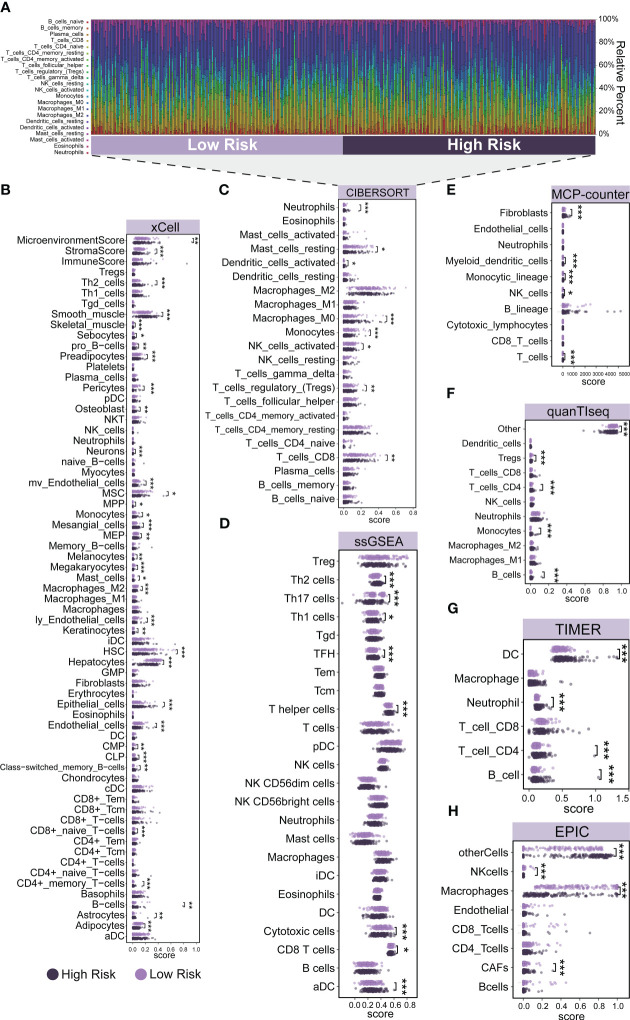
Immune cell infiltration patterns in different risk groups. **(A)** The relative percentage of 22 immune cells in the high-risk and low-risk groups. **(B–H)** Differences in immune cell infiltration between the high-risk and low-risk groups. **(B)** xCell, **(C)** CIBERSORT, **(D)** ssGSEA, **(E)** MCP-counter, **(F)** quanTIseq, **(G)** TIMER, and **(H)** EPIC. Significance signs: *p< 0.05; **p< 0.01; ***p< 0.001.

To investigate the role of risk scores consisting of three prognostic genes in the LIHC tumor microenvironment, we evaluated the immune cell score of each LIHC sample using seven algorithms: xCell, CIBERSORT, ssGSEA, MCP-counter, quanTIseq, TIMER, and EPIC. A more detailed and diverse uniform access to bulk RNA sequencing data is available to assess the immune cell scores of each hepatocellular liver cancer sample. This allows a comparative analysis of immune cell infiltration between the high-risk and low-risk groups. The stacked histogram of **Figure 3A** shows the relative percentages of 22 immune cells in the high-risk and low-risk groups obtained by the CIBERSORT algorithm. **Figures 3B–H** shows the differences in most immune cell infiltration between the high- and low-risk groups. We observed that CD4+ T cells infiltrated at higher levels in the high-risk group than in the low-risk group, where the results of the CIBERSORT algorithm showed higher levels of Tregs infiltration in the high-risk group, the results of the TIMER and quanTIseq algorithms showed higher levels of CD4+ T cells or Tregs infiltration in the high-risk group, and the results of the ssGSEA algorithm indicated Th1, Th2, and T helper cells had higher infiltration levels in the high-risk group, and the results of the xCell algorithm demonstrated higher infiltration levels of Th2 and CD4+ memory T cells in the high-risk group. As for CD8+ T cells, by ssGSEA, CIBERSORT, and xCell algorithms, we found that their infiltration levels were lower in the high-risk group than in the low-risk group, where the xCell algorithm showed lower infiltration levels of CD8+ naive T cells in the high-risk group. For B cells, we observed that their infiltration levels were higher in the high-risk group than in the low-risk group by the xCell, TIMER, and quanTIseq algorithms. For Neutrophils, the results of TIMER and CIBERSORT algorithms showed higher levels of infiltration in the high-risk group. For NK cells, we observed a lower level of infiltration in the high-risk group by the EPIC and CIBERSORT algorithms. For Macrophages, we observed a lower level of infiltration in the high-risk group by EPIC, CIBERSORT, and xCell algorithms. For DC, we observed a higher level of infiltration in the high-risk group by TIMER, CIBERSORT, and ssGSEA algorithms. In addition, we performed a validation of immune cell infiltration in an independent dataset (ICGC-LIRI) (**Supplementary Figure 3A**). Using the CIBERSORT algorithm, we observed higher infiltration levels of M0 Macrophages, Tregs, Dendritic cells resting, and T cells CD4 memory activated in the high-risk group. This is in general agreement with the results obtained from the TCGA dataset.

### 3.6 Tumor-associated signaling pathway analysis and stemness index (mRNAsi) analysis

**Figure 4:**
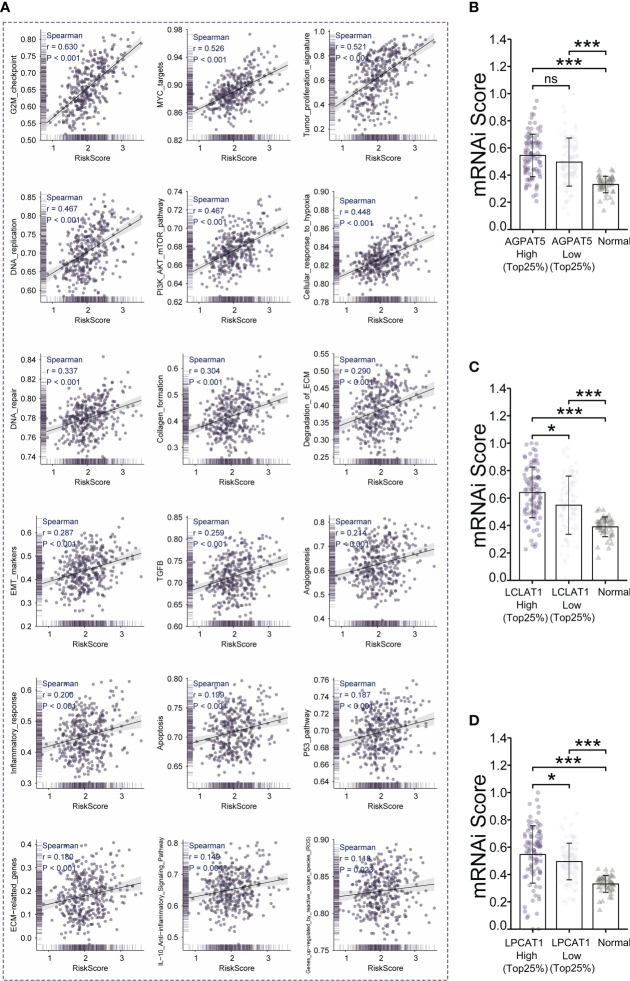
Tumor-associated signaling pathway analysis and stemness index (mRNAsi) analysis. **(A)** Analysis of the correlation between risk scores and 18 tumor signaling pathways. **(B–D)** Comparison of mRNAsi in the high- and low-gene-expression (*AGPAT5*, *LCLAT1*, and *LPCAT1*, respectively) groups. Significance signs: ns, p ≥ 0.05; *p< 0.05; **p< 0.01; ***p< 0.001.

We selected genes functioning in common tumor-associated pathways (Cellular_response_to_hypoxia, Tumor_proliferation_signature, EMT_markers, ECM-relatted_genes, Angiogenesis, Apoptosis, DNA_repair, G2M_checkpoint, Inflammatory_response, PI3K_AKT_mTOR_pathway, P53_pathway, MYC_targets, TGFB, IL-10_Anti-inflammatory_Signaling_Pathway, Genes_up-regulated_by_reactive_oxigen_species_(ROS), DNA_replication, Collagen_formation, and Degradation_of_ECM) and analyzed them using the R package “GSVA”, selecting the parameter method=“ssgsea”. We calculated the enrichment scores of each sample in each pathway in turn, and finally determined the correlation between risk score and pathway scores using Spearman’s rank-order correlation. The results showed a strong positive correlation between risk scores and all tumor-related pathways we selected (**Figure 4A**). Based on the RNAseq data of the TCGA-LIHC dataset, we used the OCLR algorithm to calculate the mRNAsi (degree of stemness) for each sample (35). We found that the mRNAsi was higher in the high-*LCLAT1*-expression and high-*LPCAT1*-expression groups relative to that in the respective low-expression groups (Top25%) (*p*< 0.05 and *p*< 0.05, respectively) (**Figures 4C, D**). The high-*AGPAT5*-expression group (Top25%) also displayed a trend for a greater degree of stemness compared with the respective low-expression group (*p* = 0.29) (**Figure 4B**). And a higher stemness index was associated with biological activity in cancer stem cells and larger dedifferentiation of the tumor.

### 3.7 Relationship between gene expression and clinical characteristics

**Figure 5:**
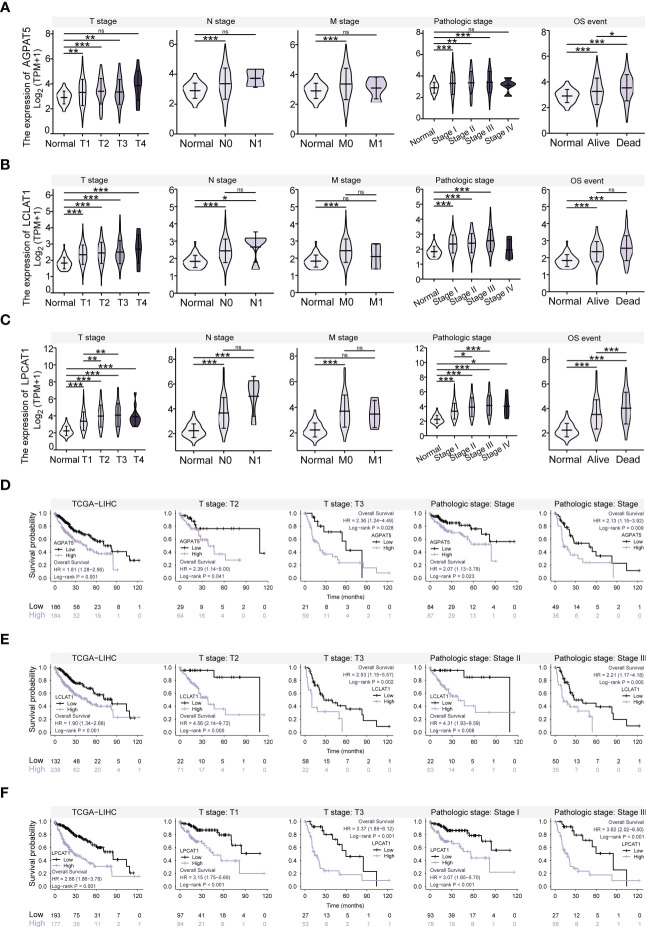
Relationship between Gene Expression and Clinical Characteristics, and Kaplan-Meier Survival Analysis. **(A)** Expression of *AGPAT5* gene in different TNM stages, pathological stages, and survival subgroups compared with that in normal samples. **(B)** Expression of *LCLAT1* gene in different TNM stages, pathological stages, and survival subgroups compared with that in normal samples. **(C)** Expression of *LPCAT1* gene in different TNM stages, pathological stages, and survival subgroups compared with that in normal samples. **(D)** Survival curves for *AGPAT5* high and low expression groups, for all patients, T2 stage, T3 stage, pathologic stage I, and pathologic stage III, respectively. **(E)** Survival curves for *LCLAT1* high and low expression groups, for all patients, T2 stage, T3 stage, pathologic stage II, and pathologic stage III, respectively. **(F)** Survival curves for *LPCAT1* high and low expression groups, for all patients, T1 stage, T3 stage, pathologic stage I, and pathologic stage III, respectively. Significance signs: ns, p ≥ 0.05; *p< 0.05; **p< 0.01; ***p< 0.001.

We explored the expression levels of *AGPAT5*, *LCLAT1*, and *LPCAT1*, the three core genes of the prognostic model, in normal and pan-cancer tissues based on XENA-TCGA-GTEx datasets. We found that *AGPAT5*, *LCLAT1*, and *LPCAT1* were highly expressed in most tumors (**Supplementary Figures 3B–D**). A search of the published literature revealed a scarcity of information regarding the role of the three signature genes in HCC. We next investigated the link between gene expression and clinical characteristics using the Kruskal-Wallis test. The results indicated that the expression of the three genes was higher in all the samples from different TNM stage subgroups and pathologic stage subgroups than in the normal samples (**Figures 5A–C**). *LPCAT1* expression was higher in stages T2 and T3 than in the T1 stage and was also upregulated in pathologic stages II and III compared with pathologic stage I. Additionally, *AGPAT5* and *LPCAT1* were more highly expressed in the Dead subgroup than in the Alive subgroup.

### 3.8 Kaplan-Meier survival analysis relating to *AGPAT5*, *LCLAT1*, and *LPCAT1* expression

Additionally, we conducted a Kaplan-Meier survival analysis relating to the expression levels of *AGPAT5*, *LCLAT1*, and *LPCAT1*. The optimal cut-off for continuous gene expression data was determined using the “surv_cutpoint” function in the R package “survminer” and the expression data were separated into high- and low-expression groups. OS was selected as the prognostic type. The results of the analysis showed that the high-expression group had a shorter OS than the low-expression group (*p*< 0.05) (**Figures 5D–F**). We also evaluated the prognostic value of these genes in the different clinical characteristic subgroups and found that the groups with high expression of *AGPAT5*, *LCLAT1*, and *LPCAT1* had shorter OS than the groups with low expression, regardless of the T stage or pathological stage (*p*< 0.05).

### 3.9 Gene mutations (*AGPAT5*, *LCLAT1*, and *LPCAT1*) in HCC

**Figure 6:**
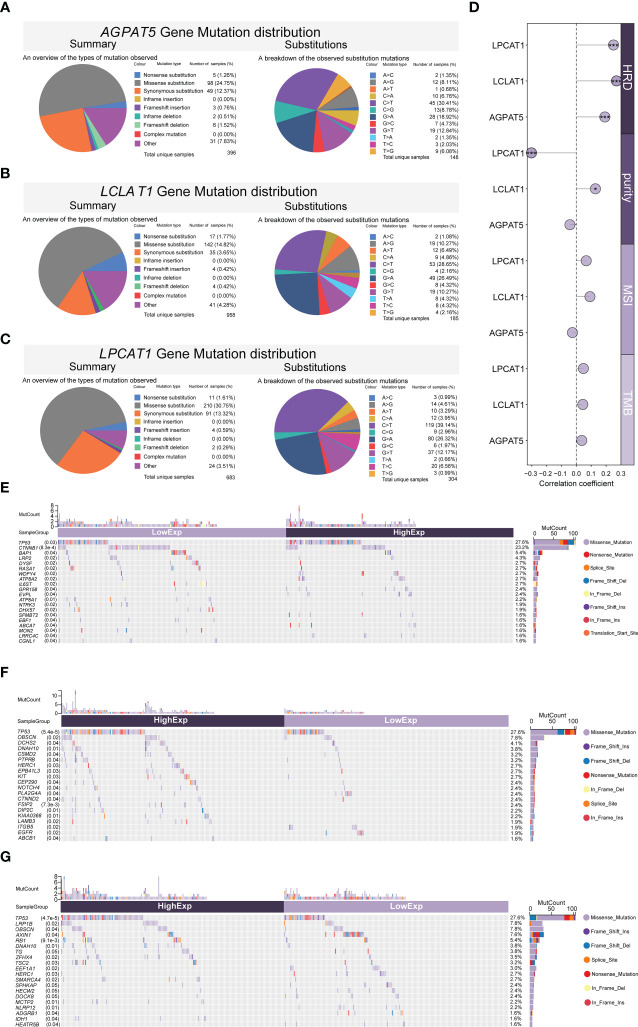
Mutations of genes (*AGPAT5*, *LCLAT1*, and *LPCAT1*) in HCC. **(A–C)** Evaluation of *AGPAT5* **(A)**, *LCLAT1* **(B)**, and *LPCAT1* **(C)** mutation types (COSMIC database). **(D)** Pearson correlations between genomic heterogeneity indicators with gene expression. **(E–G)** Differences in the somatic mutation landscape between high- and low-gene-expression (*AGPAT5*, *LCLAT1*, and *LPCAT1*, respectively) groups. Significance signs: *p< 0.05; **p< 0.01; ***p< 0.001.

We mined the COSMIC database and evaluated the mutation types in the *AGPAT5*, *LCLAT1*, and *LPCAT1* genes. For better visualization, the mutation types are depicted as pie charts. For *AGPAT5*, missense substitutions were found in approximately 24.75% of the samples and synonymous substitutions in 12.37% of the samples. C>T was the most common substitution mutation (30.41%), followed by G>A (18.92%) and G>T (12.84%) (**Figure 6A**). For *LCLAT1*, missense substitutions occurred in approximately 14.82% of the samples, with C>T again being the most frequently detected substitution mutation (28.65%), followed by G>A (26.49%), G>T (10.27%), and A>G (10.27%) (**Figure 6B**). For *LPCAT1*, missense substitutions occurred in approximately 30.75% of the samples and synonymous substitutions in 13.32% of the samples. C>T was the most commonly observed substitution mutation (39.14%), followed by G>A (26.32%) and G>T (12.17%) (**Figure 6C**). We also calculated Pearson correlations of genomic heterogeneity indicators with gene expression, and we observed that expression of *AGPAT5*, *LCLAT1*, and *LPCAT1* were significantly positively correlated with HRD. expression of *LCLAT1* was significantly positively correlated with purity, and expression of *LPCAT1* was significantly negatively correlated with purity (**Figure 6D**). In addition, we compared the somatic mutation landscape between the three high- and low-gene-expression groups (*AGPAT5*, *LCLAT1*, and *LPCAT1*) in the TCGA-LIHC dataset. The most common differentially mutated genes between the high-*AGPAT5*-expression and low-*AGPAT5*-expression groups were *TP53* (*p* = 0.03), *CTNNB1* (*p* = 0.00083), *BAP1* (*p* = 0.04), *LRP2* (*p* = 0.02), and *DYSF* (*p* = 0.02) (**Figure 6E**). The most common differentially mutated genes between the high-*LCLAT1*-expression and low-*LCLAT1*-expression groups were *TP53* (*p* = 0.000054), *OBSCN* (*p* = 0.02), *DCHS2* (*p* = 0.04), *DNAH10* (*p* = 0.01), and *CSMD2* (*p* = 0.04) (**Figure 6F**). The most common differentially mutated genes between the high-*LPCAT1*-expression and low-*LPCAT1*-expression groups were *TP53* (*p* = 0.000047), *LRP1B* (*p* = 0.02), *OBSCN* (*p* = 0.04), *AXIN1* (*p* = 0.04), and *RB1* (*p* = 0.0091) (**Figure 6G**).

### 3.10 Analysis of immune checkpoint differences and potential immunotherapeutic response

**Figure 7:**
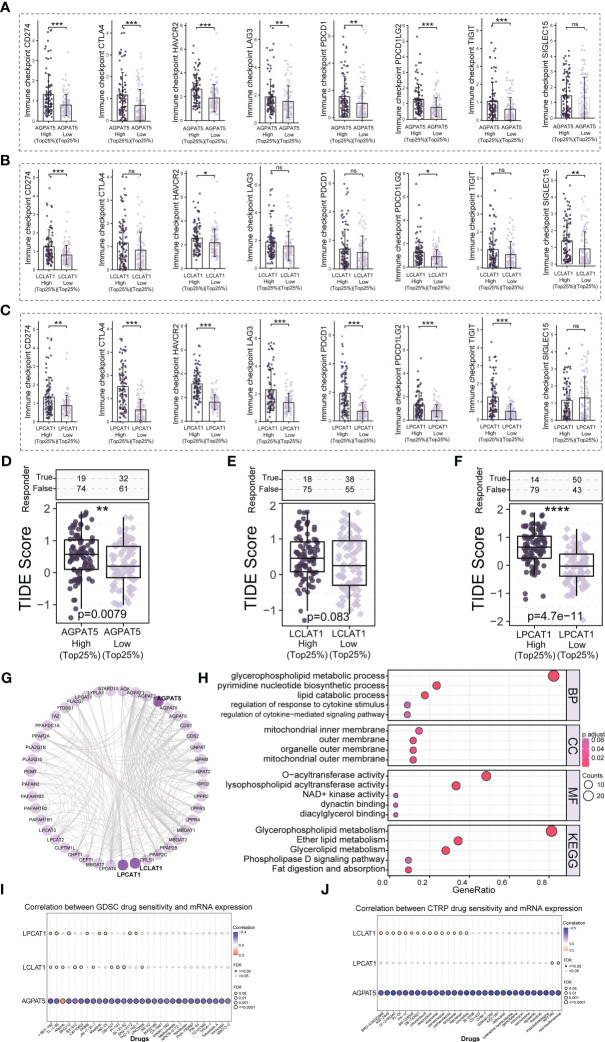
Potential immunotherapeutic response and drug sensitivity analysis. **(A–C)** The expression of eight immune checkpoint-related genes in the high- and low-gene-expression (*AGPAT5*, *LCLAT1*, and *LPCAT1*) groups. **(D–F)** Comparison of TIDE scores in the high- and low-gene-expression (*AGPAT5*, *LCLAT1*, and *LPCAT1*) groups. **(G)** Network diagram of AGPAT5-, LCLAT1-, and LPCAT1-interacting proteins. **(H)** GO and KEGG enrichment analysis of AGPAT5-, LCLAT1-, and LPCAT1-interacting proteins. **(I, J)** Bubble plots of the correlation between *AGPAT5*, *LCLAT1*, and *LPCAT1* mRNA expression and drug IC50 values. Significance signs: ns, p ≥ 0.05; *p< 0.05; **p< 0.01; ***p< 0.001.

We analyzed the expression of eight common immune checkpoint-related genes (*CD274*, *CTLA4*, *HAVCR2*, *LAG3*, *PDCD1*, *PDCD1LG2*, *TIGIT*, and *SIGLEC15*) in different subgroups (**Figures 7A–C**) and could see that most of the immune checkpoint-related genes had higher expression levels in the *AGPAT5*, *LCLAT1*, and *LPCAT1* high expression groups. We also predicted the potential immunotherapeutic response based on the TIDE algorithm (36). When the TIDE score was high, immune checkpoint blockade (ICB) efficacy was poor and survival after receiving ICB therapy was short. Our calculations showed that the TIDE score was significantly higher in the high-*AGPAT5*-expression and high-*LPCAT1*-expression groups (Top25%) (p = 0.0079 and p = 4.7e-11, respectively) (**Figures 7D, F**). The high-*LCLAT1*-expression group (Top25%) also exhibited a trend of higher TIDE scores (p = 0.083) (**Figure 7E**).

### 3.11 PPI network construction and drug sensitivity analysis

To identify the potential AGPAT5, LCLAT1, and LPCAT1 interacting proteins, we constructed a PPI network using the STRING database (interaction score >0.7) and further visualized the resulting network using the R packages “igraph” and “ggraph” (**Figure 7G**). We combined the data for AGPAT5, LCLAT1, and LPCAT1 interacting proteins and performed GO and KEGG enrichment analysis. The results indicated that, in biological processes, AGPAT5-, LCLAT1, and LPCAT1 interacting proteins were mainly enriched in glycerophospholipid metabolic process, pyrimidine nucleotide biosynthetic process, and lipid catabolic process. For cellular components, AGPAT5, LCLAT1, and LPCAT1 interacting proteins were mainly associated with mitochondrial inner membrane, outer membrane, organelle outer membrane, and mitochondrial outer membrane. In molecular function, meanwhile, the interacting proteins were mainly enriched in *O*-acyltransferase activity and lysophospholipid acyltransferase activity. Regarding KEGG pathways, AGPAT5, LCLAT1, and LPCAT1 interacting proteins were primarily enriched in Glycerophospholipid metabolism, Ether lipid metabolism, and Glycerolipid metabolism (**Figure 7H**). We also explored the correlation between the mRNA expression of *AGPAT5*, *LCLAT1*, and *LPCAT1* and drug sensitivity (IC50 values) (**Figures 7I, J**). The results showed that *AGPAT5* was negatively regulated by various drugs or small molecule targets, such as I-BET-762, FK866, NPK76-II-72-1, LY-2183240, vincristine, BI-2536, GSK461364, KX2-391, etc., while *LCLAT1* was negatively regulated by Afatinib. *LPCAT1* was negatively regulated by KPT185 and necrosulfonamide. These results provide a possible strategy for clinical treatment of abnormal expression of *AGPAT5*, *LCLAT1*, and *LPCAT1* in patients with LIHC.

### 3.12 Preliminary validation of AGPAT5, LCLAT1, and LPCAT1

**Figure 8:**
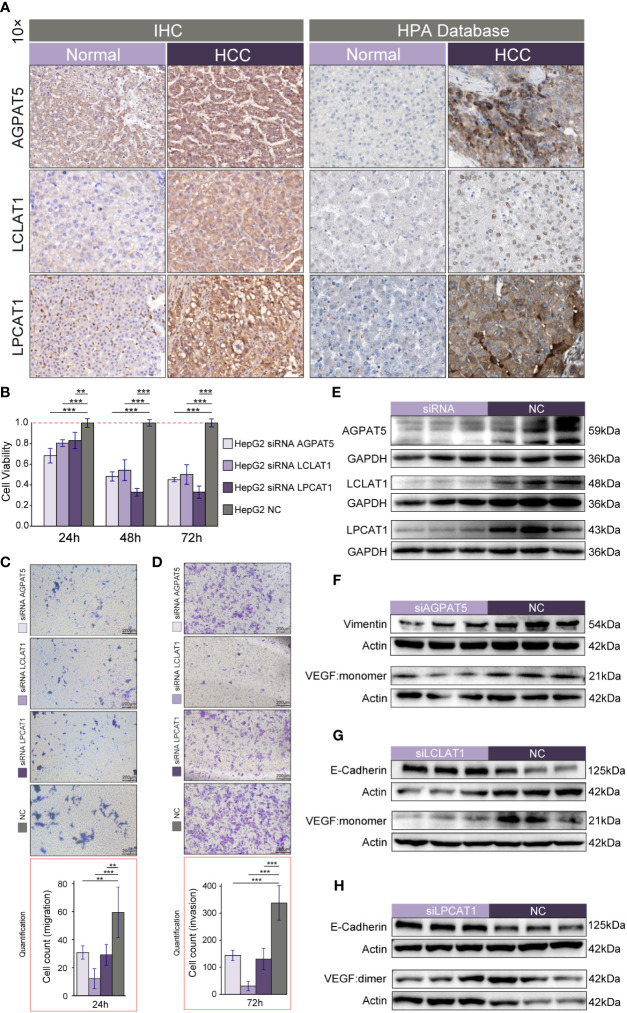
Preliminary validation of *AGPAT5*, *LCLAT1*, and *LPCAT1*. **(A)** Immunohistochemistry of HCC tumor tissues and adjacent normal tissues. **(B–D)** CCK-8 assay **(B)**, migration assay **(C)**, and invasion assay **(D)** after knockdown of *AGPAT5*, *LCLAT1*, and *LPCAT1* in HepG2 using siRNA and non-targeted control siRNA, respectively. **(E)** Western blotting validation of knockdown efficacy after transfection of HepG2 using siRNA and non-targeting control siRNA. **(F)** Validation of common tumor-associated protein markers after knockdown of *AGPAT5*. **(G)** Validation of common tumor-associated protein markers after knockdown of *LCLAT1*. **(H)** Validation of common tumor-associated protein markers after knockdown of *LPCAT1*. Representative images are shown. Magnification 100x; scale bar: 200 μm. Significance signs: *p< 0.05; **p< 0.01; ***p< 0.001.

To verify the protein expression levels of *AGPAT5*, *LCLAT1*, and *LPCAT1*, we performed immunohistochemistry. The results of tissue samples showed that AGPAT5, LCLAT1, and LPCAT1 were significantly upregulated in HCC tumor tissues than in adjacent normal tissues (**Figure 8A**). Similarly, immunohistochemistry from HPA Database further validated our results (**Figure 8A**) (**Supplementary Figure 1**). To further explore the functions that *AGPAT5*, *LCLAT1*, and *LPCAT1* may exercise in the development of HCC. First, we explored the expression of *AGPAT5*, *LCLAT1*, and *LPCAT1* in common hepatocellular carcinoma cell lines by data mining in the CCLE database. We found that the transcript levels of these three genes were highly expressed in HepG2 cell lines (**Supplementary Figures 4A–C**). Therefore, we used siRNA and non-targeting control siRNA to knock down *AGPAT5*, *LCLAT1*, and *LPCAT1* in HepG2, respectively. CCK8 experiments showed that knocking down *AGPAT5*, *LCLAT1* and *LPCAT1* could significantly inhibit the proliferation of HepG2 cells (**Figure 8B**). Furthermore, by performing Transwell experiments, we observed that silencing of *AGPAT5*, *LCLAT1*, and *LPCAT1* effectively inhibited the migration (**Figure 8C**) and invasion (**Figure 8D**) ability of HepG2 cells. In short, the knockdown of *AGPAT5*, *LCLAT1*, and *LPCAT1* effectively attenuated the proliferation, migration, and invasion of HepG2 cells. We observed by Western blotting that the protein levels of AGPAT5, LCLAT1, and LPCAT1 were significantly downregulated after siRNA transfection compared to NC-siRNA-transfected cells (**Figure 8E**). In addition, we verified common tumor-associated protein markers and found that after the knockdown of *AGPAT5* (**Figure 8F**) (**Supplementary Figure 4D**), their VEGF levels were significantly reduced, along with a significant trend of Vimentin reduction. After the knockdown of *LCLAT1* (**Figure 8G**) (**Supplementary Figure 4E**) or *LPCAT1* (**Figure 8H**) (**Supplementary Figure 4F**), the results showed a significant increase in the protein level of E-Cadherin and a significant decrease in the level of VEGF.

## 4 Discussion

Many methods are available for staging HCC, such as BCLC staging (37), AJCC-TNM (38), CLIP (39), Tokyo systems (40), and HKLC (41). A study comparing 11 HCC staging systems (42) found that CLIP can be used for a more accurate prognosis before treatment, while the BCLC and HK systems (43) are better for treatment selection. However, these staging and scoring methods have a limited ability to accurately predict the survival of HCC patients and require updating.

The clinical and pathological implications of the molecular staging of HCC are mostly at the research and demonstration stage. Accordingly, it is important to identify prognostic biomarkers and to optimally stratify patients with HCC to implement more accurate diagnosis and treatment for the control of this disease. Therefore, in this study, we developed and validated a risk prediction model based on the *GPAT*/*AGPAT* gene family to better stratify patients with HCC. The model was externally validated using the ICGC-LIRI dataset. Patients in the high-risk group had a lower survival rate and a higher risk score than those in the low-risk group. Univariate and multivariate Cox regression analysis demonstrated that the risk score was a meaningful prognostic indicator and an independent predictor of OS in HCC.

The expression of AGPAT isoforms has been shown to enhance tumor cell proliferation and drug resistance and is associated with an increased risk of tumorigenesis or the development of aggressive phenotypes in a variety of cancers (9). Therefore, we built a machine-learning model with LASSO regression to identify the variables in this gene family that is most associated with OS in HCC and to reduces the effect of multicollinearity (44). This reduced the complexity of the model and improved its predictive accuracy. A signature consisting of three genes of the *GPAT*/*AGPAT* family (*AGPAT5*, *LCLAT1*, and *LPCAT1*) showed high specificity and sensitivity in predicting OS in patients with HCC. The expression of these three core genes was negatively correlated with a good prognosis. We also established a nomogram that combined risk score and TNM staging that predicted with high accuracy the survival at 1, 3, and 5 years of patients with HCC, with AUC values of 0.807, 0.806, and 0.795, respectively. The risk score improved the reliability of the nomogram and could serve as a guide for decision-making in the clinic.

We found that *AGPAT5*, *LCLAT1*, and *LPCAT1* expression is commonly upregulated in HCC tissues, while patients with high levels of expression of these genes have a worse prognosis. AGPAT5 was only detected in mitochondria (45). Phosphatidic acid (PA) synthesis is catalyzed by AGPATs, and PA has been demonstrated to promote tumor cell survival, proliferation, and metastasis. However, beyond PA synthesis, the specific physiological role of AGPAT5 is currently unknown. AGPAT5 may have a crucial function in mitochondrial fusion and division (9). In turn, the results of recent studies suggest that mitochondrial membrane fusion-mediated increases in oxidative phosphorylation and NADH/NAD+ metabolism contribute to tumor immortalization (46). It has also been reported that miR-26, which is downregulated by estrogen in breast cancer cell lines, can directly target *AGPAT5 via* its 3′UTR (47). The LCLAT1 protein is predicted to be associated with phosphatidylinositol acyl-chain remodeling and is localized to the cytoplasm and endoplasmic reticulum. Recently, *LCLAT1* was identified as a partner gene for *ALK* in a patient with non-small cell lung cancer (NSCLC). The patient showed a partial response to crizotinib (48). Moreover, high expression of the *LPCAT1* gene has been implicated in the pathology of lung adenocarcinoma (49), breast cancer (50), prostate cancer (51), esophageal squamous cell carcinoma (52), and HCC (53), among other cancers. Accordingly, *LPCAT1* has the potential as a therapeutic target for the inhibition of HCC progression (54) as well as a marker for the prognosis of tumor patients. However, whether *AGPAT5* and *LCLAT1* have a clear role in HCC remains to be determined.

In this study, we screened these three differential genes with prognostic value by combing through public databases. Meanwhile, we further confirmed by IHC that the protein levels of AGPAT5, LCLAT1, and LPCAT1 were significantly upregulated in HCC tissues compared with adjacent normal tissues, which was consistent with the transcript levels and also with the results of the HPA Database. Furthermore, we confirmed the ability of major members of this gene family to participate in tumor cell proliferation, migration, and invasion by silencing *AGPAT5*, *LCLAT1*, and *LPCAT1*, respectively, in HepG2 cell lines.

A series of biomarkers of the Epithelial-mesenchymal transition (EMT) process is used in the diagnosis and prognosis of several types of tumors. reduced E-cadherin expression levels are an important marker event for the development of EMT, and E-Cadherin deficiency is strongly associated with EMT in a variety of tumors (55, 56). Also, E-cadherin expression was negatively correlated with tumor cell motility and invasive behavior as well as metastasis in cancer patients (56). When we knocked down both *LCLAT1* and *LPCAT1* in hepatocellular carcinoma cell lines, we could observe a significant increase in the protein level of E-cadherin. si-LPCAT1 results again validated the reported findings (57), but si-LCLAT1 results were the first ones we found. In addition, we observed that a significant reduction in the protein level of VEGF was observed after knocking down respectively three genes of prognostic value in this gene family. *VEGFA* is one of the 34 most frequently reported genes in HCC (58), which encodes vascular endothelial growth factor A, a heparin-binding protein that induces vascular endothelial cell proliferation and migration and is required for physiological and pathological angiogenesis (59). This gene is upregulated in many known tumors and its expression plays an important role in tumor progression (60, 61). Targeted therapy of VEGFA as an innovative treatment in oncology, the VEGFA inhibitor bevacizumab has been used as a first-line treatment for metastatic colorectal cancer since 2004 (59, 62). In the treatment of hepatocellular carcinoma, the combination of atezolizumab and bevacizumab has been shown to improve overall survival relative to sorafenib (63, 64). Given its important role in tumor angiogenesis, targeted therapy of VEGF signaling has emerged as one of the key avenues for the development of anti-angiogenic therapies. Our findings provide new ideas for the co-application of anti-angiogenic therapies. In addition, our oncological phenotypic experiments lay the foundation for further investigation of the potential functions of *AGPAT5* and *LCLAT1* in HCC. Therefore, we consider these three members of the *GPAT*/*AGPAT* gene family (*AGPAT5*, *LCLAT1*, and *LPCAT1*) to be valued prognostic biomarkers and potential therapeutic targets for HCC patients.

This study had several limitations. First, as only relatively few HCC datasets containing prognostic information are currently available, we only used the ICGC-LIRI dataset for external validation. Second, we only performed common tumor phenotype experiments, and we did not perform Bulk sequencing on knockdown cell lines, so the more specific downstream pathways of *AGPAT5*, *LCLAT1*, and *LPCAT1* and their biological mechanisms still need to be investigated in depth, which will be further explored in our future work. In conclusion, we developed a *GPAT*/*AGPAT* gene family-related risk model for predicting the prognosis of patients with HCC, as well as a predictive nomogram for determining the prognosis of patients with HCC and for guiding their individualized treatment, which can be used to identify LIHC patients at high risk of death and provide a reference for early clinical intervention to better improve their prognosis. We also compared the immune cell infiltration in different risk populations by multiple cutting-edge algorithms. We also performed a comprehensive analysis of the three core genes of the prognostic model (*AGPAT5*, *LCLAT1*, and *LPCAT1*), involving relevant signaling pathways, mRNAi, clinical relevance, survival, mutations, ICB responses, and interacting proteins. Finally, we performed preliminary validation of the differential expression, oncological phenotypes, and potential downstream pathways of three members of the *GPAT*/*AGPAT* gene family (*AGPAT5*, *LCLAT1*, and *LPCAT1*) by IHC, CCK-8, Transwell assays, and Western blotting. Our findings enhance the understanding of the potential biological functions of the *GPAT*/*AGPAT* gene family and provide a reference for exploring prognostic biomarkers and individualized therapy for HCC.

## Data availability statement

The original contributions presented in the study are included in the article/**Supplementary Material** Further inquiries can be directed to the corresponding authors.

## Author contributions

Conception and design: ZP, YL. Administrative support: ZP. Provision of study materials: YL. Data collection and assembly: PW, RW, YX, WO, YY, SZ. Data analysis and interpretation: PW, RW, YX. Manuscript writing: All authors. Final approval of the manuscript: All authors.

## Supplementary material

Supplementary Figure 1 Differential expression of *GPAT*/*AGPAT* gene family in patients with HCC (immunohistochemical staining data in the Human Protein Atlas [HPA] database). **(A–I)** The expression of GPAT3, AGPAT1, AGPAT2, AGPAT4, AGPAT5, LPCAT4, LCLAT1, LPCAT1, and LPCAT2 in tumor tissues and normal liver tissues (immunohistochemical staining data for AGPAT3 were not available in the HPA database).

Supplementary Figure 2 Validation of the prediction model based on the *GPAT*/*AGPAT* gene family. **(A)** Correlation heat map of the seven genes significantly associated with overall survival. **(B–D)** Risk score distribution, risk grouping, survival outcome, and molecular expression for the prognostic model in the TCGA-LIHC validation set, TCGA-LIHC total set, and the ICGC-LIRI external validation set, respectively. **(E, F)** Univariate and multivariate Cox regression analysis identified risk score as an independent prognostic factor after the inclusion of clinical variables (ICGC-LIRI external validation dataset).

Supplementary Figure 3 Validation of the ICGC-LIRI dataset for immune cell infiltration, Pan-cancer expression analysis. **(A)** Differences in immune cell infiltration between high- and low-risk groups within the ICGC-LIRI dataset. **(B–D)** The expression levels of *AGPAT5*, *LCLAT1*, and *LPCAT1* in normal and pan-cancer tissues.

Supplementary Figure 4 Quantification for Western blotting. **(A–C)** Expression of AGPAT5, LCLAT1, and LPCAT1 in common hepatocellular carcinoma cell lines. **(D–F)** Quantification for Western blotting (for ). Data are shown as the mean ± SD of at least three independent experiments. statistical analysis was performed using unpaired t-test to compare experimental and control groups. *P< 0.05, **P< 0.01, ***P< 0.001.
