# Supplementary figures and images for: The prognostic value of the GPAT/AGPAT gene family in hepatocellular carcinoma and its role in the tumor immune microenvironment
Source: Front Immunol. 2023 Feb 10;14:1026669. doi: 10.3389/fimmu.2023.1026669 (PMC9950581; doi:10.3389/fimmu.2023.1026669)

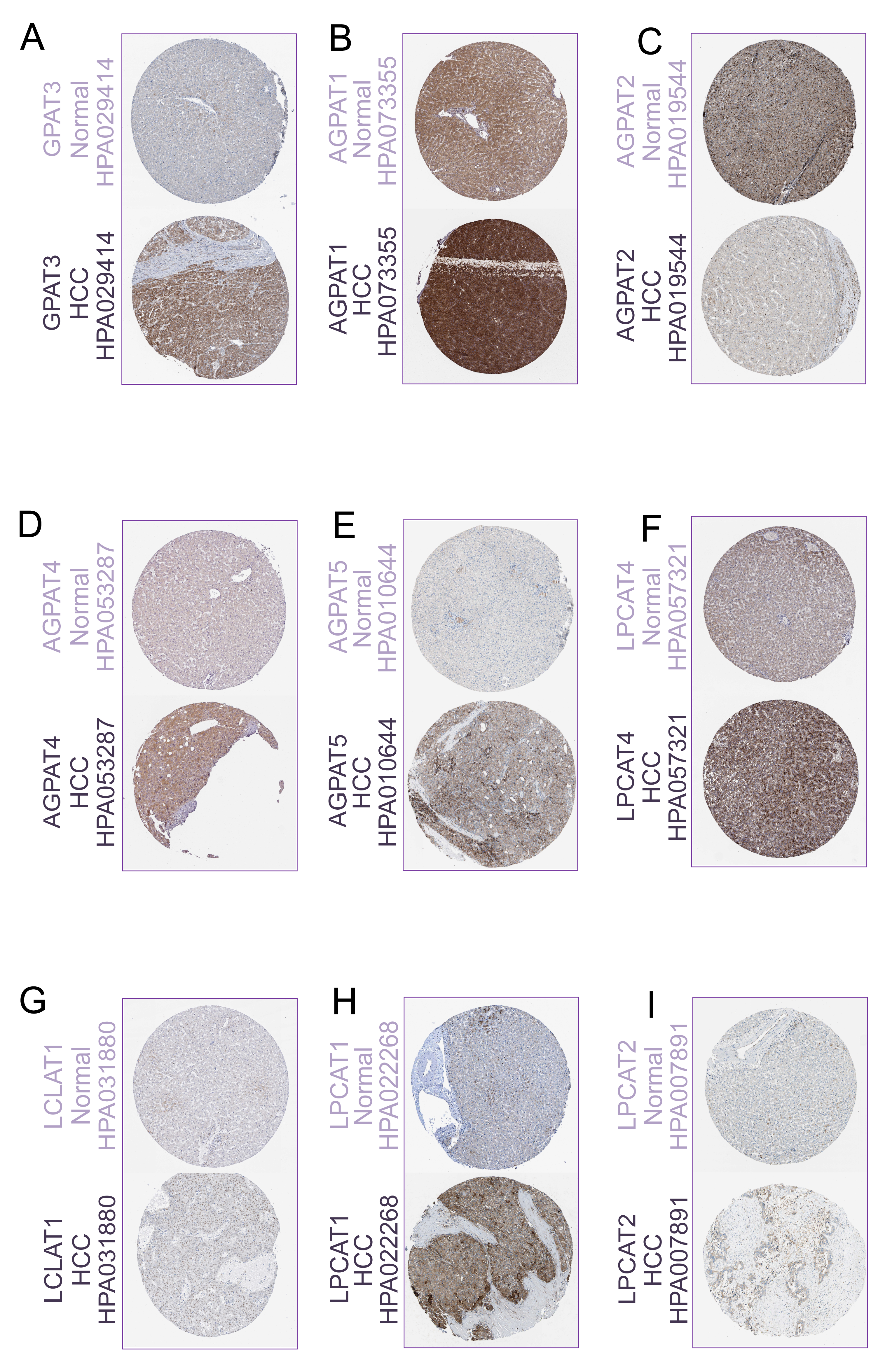

Supplement: Supplementary Figure 1 — Differential expression of GPAT/AGPAT gene family in patients with HCC (immunohistochemical staining data in the Human Protein Atlas [HPA] database). (A–I) The expression of GPAT3, AGPAT1, AGPAT2, AGPAT4, AGPAT5, LPCAT4, LCLAT1, LPCAT1, and LPCAT2 in tumor tissues and normal liver tissues (immunohistochemical staining data for AGPAT3 were not available in the HPA database). [file Image_1.tif]

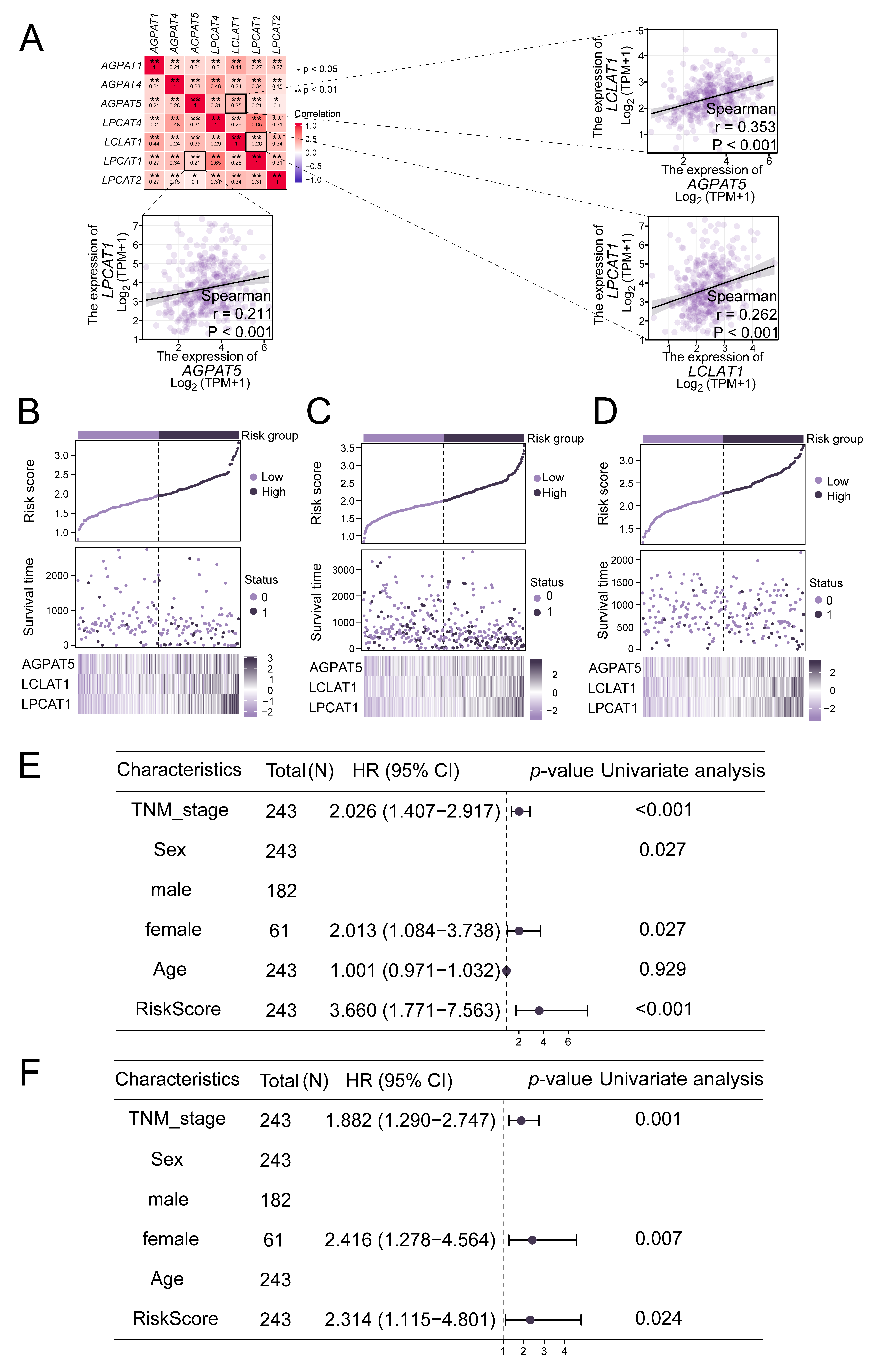

Supplement: Supplementary Figure 2 — Validation of the prediction model based on the GPAT/AGPAT gene family. (A) Correlation heat map of the seven genes significantly associated with overall survival. (B–D) Risk score distribution, risk grouping, survival outcome, and molecular expression for the prognostic model in the TCGA-LIHC validation set, TCGA-LIHC total set, and the ICGC-LIRI external validation set, respectively. (E, F) Univariate and multivariate Cox regression analysis identified risk score as an independent prognostic factor after the inclusion of clinical variables (ICGC-LIRI external validation dataset). [file Image_2.tif]

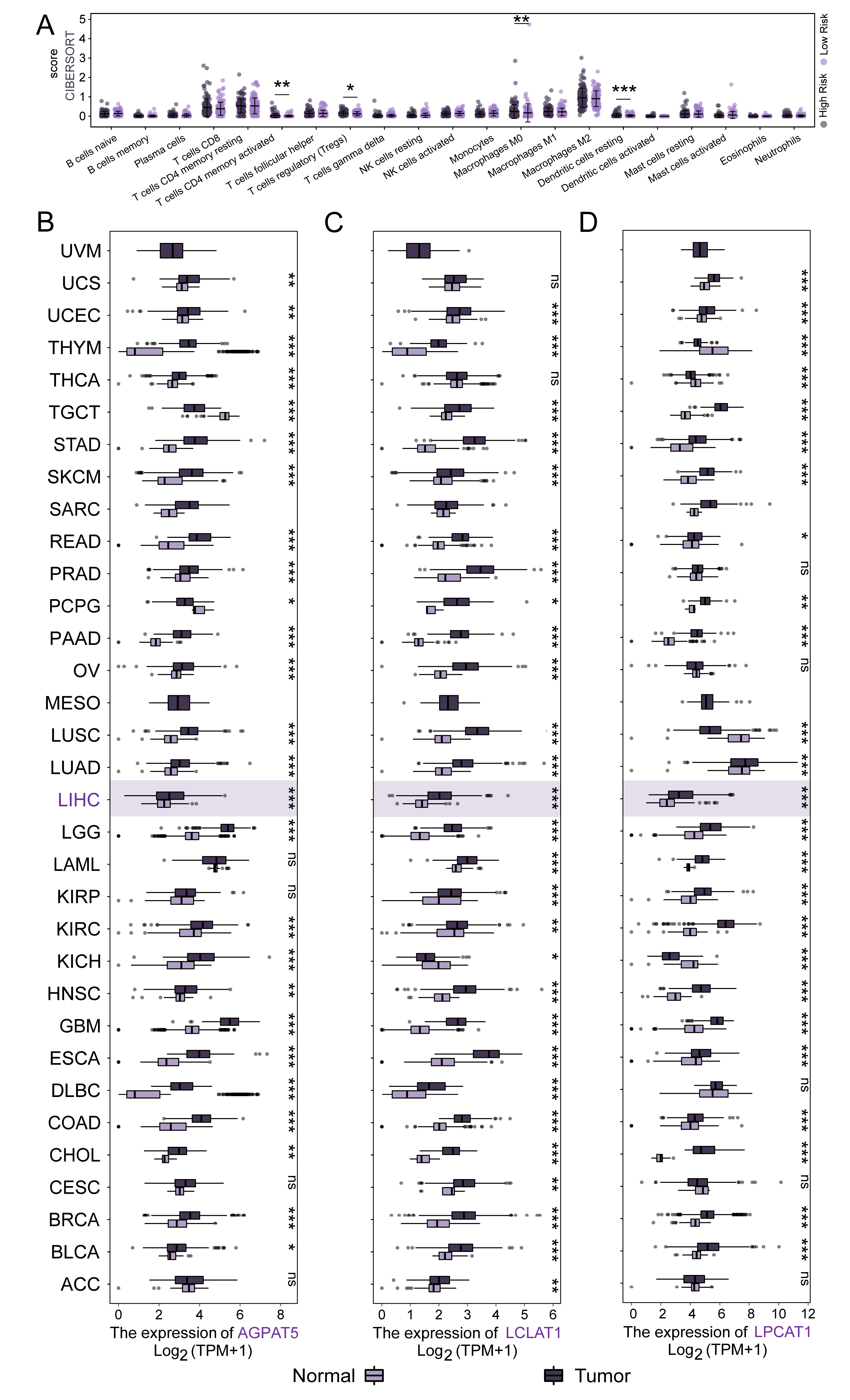

Supplement: Supplementary Figure 3 — Validation of the ICGC-LIRI dataset for immune cell infiltration, Pan-cancer expression analysis. (A) Differences in immune cell infiltration between high- and low-risk groups within the ICGC-LIRI dataset. (B–D) The expression levels of AGPAT5, LCLAT1, and LPCAT1 in normal and pan-cancer tissues. [file Image_3.tif]

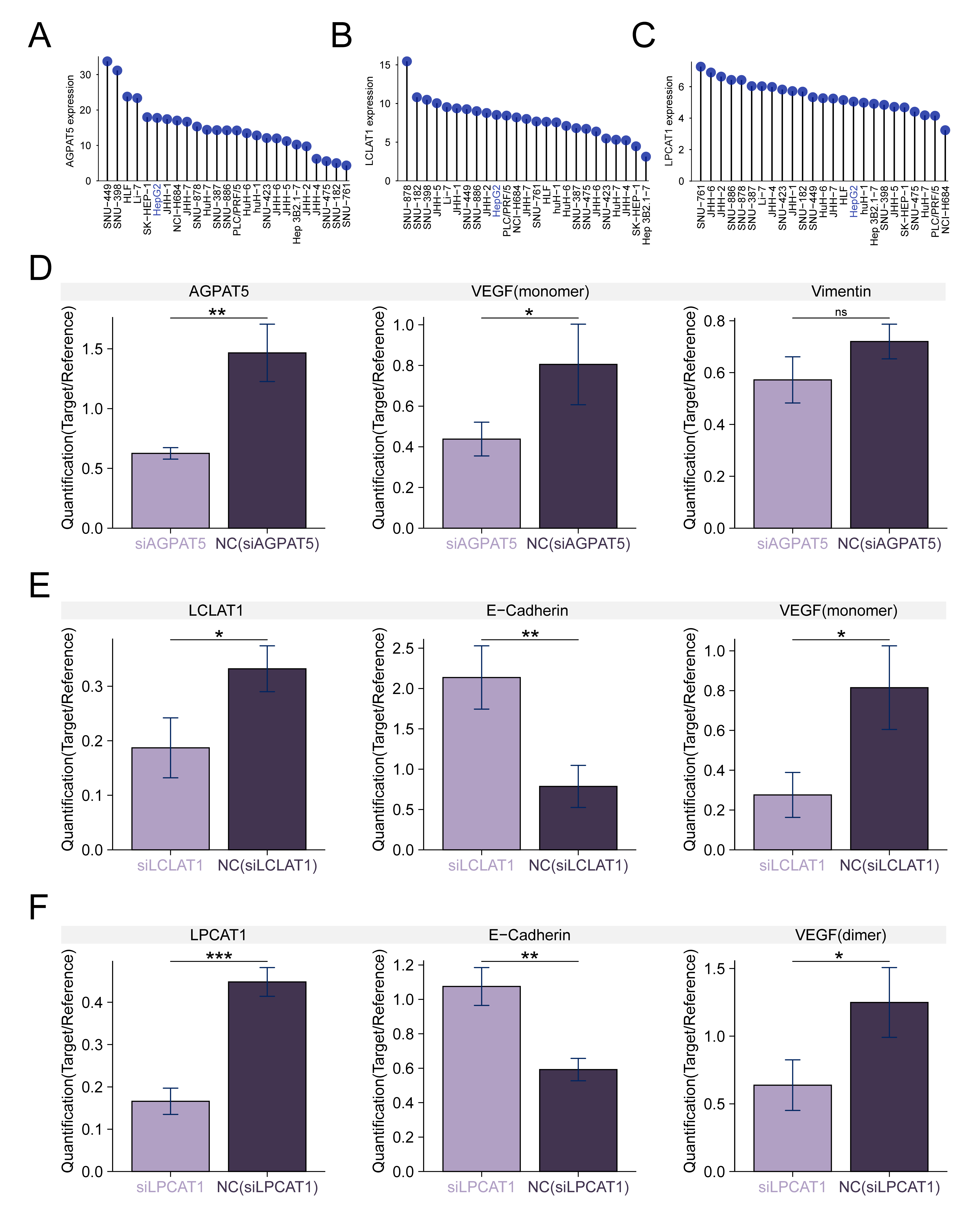

Supplement: Supplementary Figure 4 — Quantification for Western blotting. (A–C) Expression of AGPAT5, LCLAT1, and LPCAT1 in common hepatocellular carcinoma cell lines. (D–F) Quantification for Western blotting (for ). Data are shown as the mean ± SD of at least three independent experiments. statistical analysis was performed using unpaired t-test to compare experimental and control groups. *P< 0.05, **P< 0.01, ***P< 0.001. [file Image_4.tif]
